# Identification of a structurally novel BTK mutation that drives ibrutinib resistance in CLL

**DOI:** 10.18632/oncotarget.11932

**Published:** 2016-09-10

**Authors:** Shruti Sharma, Natalie Galanina, Ailin Guo, Jimmy Lee, Sabah Kadri, Charles Van Slambrouck, Bradley Long, Weige Wang, Mei Ming, Larissa V. Furtado, Jeremy P. Segal, Wendy Stock, Girish Venkataraman, Wei-Jen Tang, Pin Lu, Yue Lynn Wang

**Affiliations:** ^1^ Department of Pathology, University of Chicago, Chicago, IL 60637, USA; ^2^ Department of Medicine, University of Chicago, Chicago, IL 60637, USA; ^3^ Center for Research Informatics, University of Chicago, Chicago, IL 60637, USA; ^4^ Ben-May Department for Cancer Research, University of Chicago, Chicago, IL 60637, USA

**Keywords:** chronic lymphocytic leukemia, Richter transformation, ibrutinib resistance, *BTK*, molecular targeted therapy

## Abstract

Ibrutinib (ibr), a first-in-class Bruton tyrosine kinase (BTK) inhibitor, has demonstrated high response rates in both relapsed/refractory and treatment naïve chronic lymphocytic leukemia (CLL). However, about 25% of patients discontinue ibrutinib therapy at a median follow-up of 20 months and many patients discontinue the treatment due to leukemia progression or Richter transformation. Mutations affecting the C481 residue of BTK disrupt ibrutinib binding and have been characterized by us and others as the most common mechanism of ibrutinib resistance. Thus far, all described BTK mutations are located in its kinase domain and mutations outside this domain have never been described. Herein, we report a patient whose CLL progressed, was salvaged with ibrutinib and then relapsed. Serial analysis of samples throughout patient's clinical course identified a structurally novel mutation (*BTK*^T316A^) in the SH2 domain, but not kinase domain, of Bruton tyrosine kinase which was associated with disease relapse. Functionally, cells carrying *BTK*^T316A^ show resistance to ibrutinib at both cellular and molecular levels to a similar extent as *BTK^C481S^.* Our study lends further insight into the diverse mechanisms of ibrutinib resistance that has important implications for the development of next-generation BTK inhibitors as well as mutation detection in relapsed patients.

## INTRODUCTION

Ibrutinib (ibr), a first-in-class BTK inhibitor, has demonstrated high response rates in both relapsed/refractory and treatment naïve chronic lymphocytic leukemia (CLL) [1, 2]. However, about 25% of patients discontinue ibr therapy at a median follow-up of 20 months. Notably, 40-42% of these patients stopped the treatment as a result of disease progression [3, 4]. Among progressed patients, at least half developed Richter's transformation (RT). Treatment options for these patients are limited and outcomes are dismal with a mortality rate exceeding 75% and a median overall survival (OS) of 3 months [4]. As the use of ibr becomes more prevalent in CLL and other types of non-Hodgkin lymphoma (NHL), more patients are expected to develop resistance [5]. Thus a complete understanding of the mechanisms of ibr resistance is clinically important for the development of strategies to prevent and treat ibr-relapsed patients.

Recent studies including ours have provided some insights into ibr-resistance. Both *BTK^C481S^* and phospholipase C-γ2 (*PLCG2*) mutations have been identified [3, 6, 7]. We have demonstrated that substitution of cysteine 481 with serine in BTK resulted in loss of covalent ibr binding, restoration of *BTK* activity in the presence of ibr, subsequent reactivation of the B-cell receptor (BCR) signaling that enabled cell proliferation. These molecular and cellular events eventually lead to clinical relapse [6]. Since the first identification of *BTK*^C481S^, other *BTK* mutations (C481F/Y/R, T474I/S, and L528W) have been found in ibr refractory cases. However, the cause-and-effect relationships have not yet been established for these mutations since some of the variants were present at only 4-8% variant allele frequencies [3]. In addition, *BTK* mutations have been observed in several Richter transformed patients treated with ibr. It is currently not clear whether *BTK* mutations are related to Richter transformation. Here we describe a patient with CLL and RT who received multiple treatments including ibr. With longitudinal next-gen sequencing analysis of four samples collected throughout the disease and treatment course, we gained further insights into the mechanisms of ibr resistance that may influence the rational design of next-generation BTK inhibitors as well as mutation detection for emerging ibr resistance.

## RESULTS

### Patient clinical history and pathological characterization

**Figure 1:**
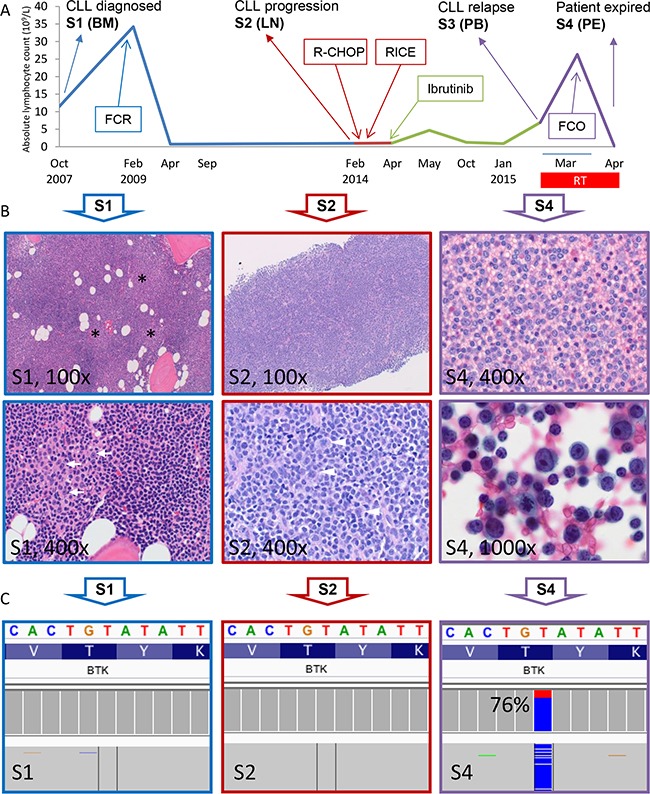
Identification of *BTK* T316A mutation in the Richter-transformed and CLL-relapsed patient **A.** Patient's absolute lymphocytosis (ALC) is plotted over disease and treatment course. The four sample collection time points, S1-S4, are shown. Blue section indicates time before CLL histologic progression; Red, time after progression; Green, time after ibr treatment, and Purple, time after RT relapse. The transient lymphocytosis following ibr treatment (green peak) is shown. BM, bone marrow; LN, lymph node; PB, peripheral blood; PE, pleural effusion. **B.** Morphologic progression of CLL to RT of large B-cell lymphoma. *S1 (100x)*, bone marrow biopsy at the time of diagnosis; proliferation centers (black stars) can be seen as vaguely pale nodular areas at low magnification. *S1 (400x)*, at high magnification occasional scattered prolymphocytes (white arrows) are seen. *S2 (100x)*, lymph node core biopsy taken at the time of CLL histologic progression. The lymph node architecture is effaced by a diffuse proliferation of small CLL cells. *S2 (400x)*, high magnification shows an increase in large atypical lymphoid cells and mitotic figures (white arrowheads). *S4 (400x)*, cell block from pleural effusion collected shortly before patient expired. Sheets of large lymphoid cells are present. *S4 (1000x)*, higher magnification shows highly atypical cells with frequent mitotic figures. **C.** Integrative Genomics Viewer (IGV) profile of *BTK*^T316A^ mutation of the 3 samples. Data presented are results sequenced using the CLL panel (See Methods).

The patient is a 57 year old woman who presented with constitutional symptoms in October 2007 (See Figure 1A for summary of her clinical history and treatment history). A complete blood count showed mild lymphocytosis (White blood cell 15 K/uL with 75% lymphocytes) with typical immuno-phenotypic features of CLL (cytogenetics/FISH not available). Bone marrow biopsy at this time showed hypercellular marrow extensively involved by CLL cells (66%) with proliferation centers (black stars, Figure 1B, top left). High magnification revealed occasional scattered prolymphocytes (white arrows, Figure 1B, bottom left). Following an 18-month observation period, she developed worsening fatigue and cytopenias (platelets 84 K/uL; marrow with 95% CLL cells) and proceeded to receive six cycles of FCR (fludarabine, cyclophosphamide and rituximab). She achieved a complete remission in September 2009. However, the disease returned in January 2014 with manifestations of abdominal discomfort and new pelvic lymphadenopathy. A core biopsy showed that the lymph node architecture is effaced by a diffuse proliferation of small CLL cells (Figure 1B, top middle). High magnification shows an increase in large atypical lymphoid cells and mitotic figures (white arrowheads) which are not associated with proliferation centers, a finding worrisome for CLL with histologic progression [S2] [16] (Figure 1B, bottom middle). Bone marrow aspirate demonstrated 17p deletion by FISH in 8% of cells. She was then treated with two chemoimmuno-regimens, but failed to improve (Figure 1A). She was not deemed a candidate for allogeneic stem cell transplantation due to persistent disease/cytopenias. Thus, ibr monotherapy was initiated in April 2014. The patient had a remarkable partial response that lasted 10 months. However, in February 2015 there was evidence of progressive disease with both worsening lymphadenopathy and lymphocytosis (91%) [S3, PB]. This prompted a change to fludarabine, cyclophosphamide and obinutuzumab regimen (FCO) in March 2015. Within a week, the patient developed a large pleural effusion. Cell block revealed sheets of transformed large lymphoid cells representing ∼40% of the total cellularity [S4] (Figure 1B top right). Higher magnification shows highly atypical cells with frequent mitotic figures consistent with RT to large B-cell lymphoma (Figure 1B bottom right). Shortly thereafter, the patient developed fever with altered mental status and subsequently expired in April 2015. Longitudinally, four samples had been collected from the patient: S1, bone marrow collected at initial diagnosis (Oct 2007); S2, lymph node core biopsy at CLL progression (Feb 2014, prior to ibr Rx); S3, peripheral blood following ibr failure (early March 2015); and S4, cell block of pleural fluid with 40% large cells (late March 2015 prior to death).

### Identification of *BTK*^T316A^ mutation in CLL samples post ibr relapse but not in the lymph node with CLL progression

To understand genetic mechanisms underlying ibr resistance developed in this patient, we sequenced the S1 (Dx) and S3 (Ibr failure) samples using Onco1K, a next-gen sequencing hybrid capture panel that detects genetic variation in 1200 cancer-related genes. Fifty-four and 56 somatic variants were identified in S1 and S3, respectively. Comparison of the two samples revealed several relapse-specific mutations in: *BTK*, *ZMYM3, MLLT6, and SDHA*. Among these, a novel *BTK* missense mutation T316A (Nucleotide c.946T>C, NM_000061) was detected in 75% of reads in S3 but not in S1. Sanger sequencing confirmed the presence and absence of this mutation in the two samples (S1).

To determine whether this mutation had emerged at the time of CLL histologic progression, we deep-sequenced S2 (Progr) in comparison to S1 (Dx) using a dedicated 17-gene CLL panel (Supplementary Table S1). With a sequencing depth of 3,700x, *BTK*^T316A^ was not detected in the progression lymph node (S2, with ∼5% large cell involvement) suggesting that most of the large cells do not carry this mutation. Pairwise comparison of mutations in the 17 genes between S1, S2, S3 and S4 was also performed (Figure 1C and Supplementary Table S2). *BTK* mutation was the only variant that is present in post ibr relapse samples (S3 and S4), while absent in pre-ibrutinib samples (S1 and S2).

### T316A is a structurally novel mutation located in the SH2 domain of BTK not directly interfering with ibr binding

**Figure 2:**
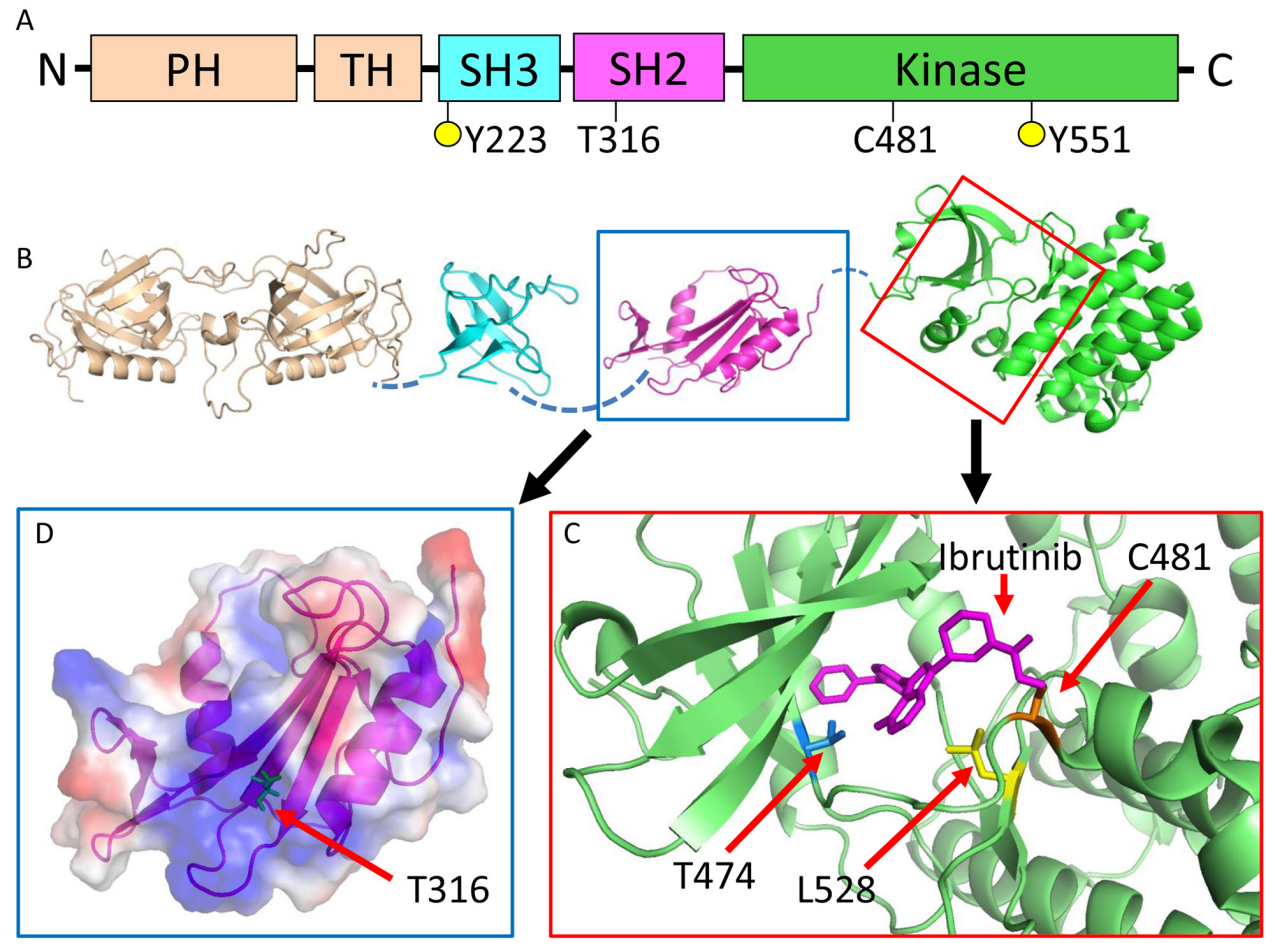
*BTK*^T316A^ is a structurally novel mutation located in the SH2 domain of BTK not directly interfering with ibr binding **A.** Schematic representation of BTK domain organization. Five domains: PH, pleckstrin homology; TH, TEC homology; SH3, SRC homology 3; SH2, SRC homology 2 and kinase domain. Y223 and Y551 are tyrosine phosphorylation sites. **B.** Structures of BTK domains. The unsolved interdomain areas are denoted by broken lines. **C.** Enlarged view of the kinase domain with ibr (magenta) binding. The three reported mutations sites are shown. C481 (orange) forms a covalent bond with ibr, which is disrupted by C481 mutations. T474 (blue) and L528 (yellow) are also located at the ibr binding pocket and mutations at these sites are expected to weaken (T474I) or hinder (L528W) ibr binding. **D.** Enlarged view of the SH2 domain with electrostatic surface potential. T316 (green) is at the center of the positively-charged binding pocket (blue area), which is predicted to interact with phosphotyrosine residues.

We hypothesized that this T316A mutation represents the molecular mechanism that confers ibr resistance in this patient. To better understand how T316A and other reported *BTK* variants involving residues C481, T474, and L528 might confer ibr resistance, we first initiated mapping of these mutations onto the available BTK domain structures [3, 6, 17–20]. Although the role of C481, which is covalently linked to ibr, is relatively well understood [6, 12], T474 and L528 have never been structurally or functionally characterized. Our structural modeling revealed that along with C481, T474 and L528 are located in the kinase domain at the ibr docking site where the mutations either directly attenuate (T474I/S) or hinder (L528W) ibr binding (Figure 2 A-C). In contrast to the kinase domain mutations, T316 is located at the center of the positively-charged binding pocket in the Src-homology 2 (SH2) domain. Unlike kinase domain mutations, T316A, at this location, does not seem to directly interfere with ibr binding. Thus, it may or may not render ibr resistance (See below and discussion).

### T316A mutation functionally confers ibr resistance at the cellular level

**Figure 3:**
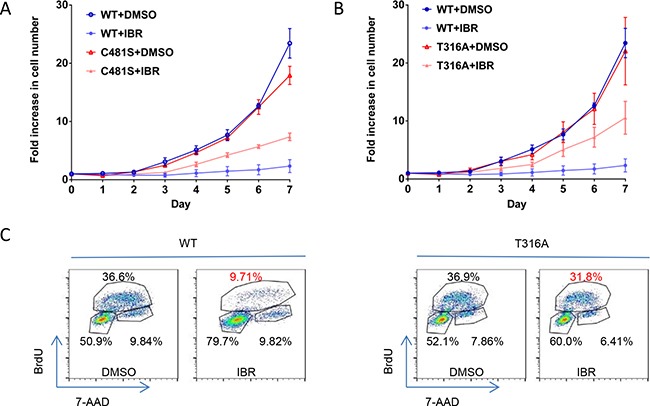
T316A mutation functionally confers ibr resistance at the cellular level **A.** Growth of TMD8 cells transfected with *BTK* T316A, C481S, and WT *BTK* constructs. The transfected cells were cultured with either 100 nM ibr or DMSO. The live cell numbers were counted daily to 7 days. The results represent four independent experiments. **B.** Cell proliferation evaluated with the BrdU incorporation assay. Cells transfected with WT *BTK* and T316A were treated with 100 nM ibr for 3 days and were labeled with 10 μM BrdU for 2 hrs.

We then resort to assessment of the functional impact of T316A mutation at the cellular levels. We constructed *BTK* wild type (WT) and *BTK*^T316A^ as well as *BTK*^C481S^ expression vectors and transfected them into an ibr-sensitive lymphoma cell line (TMD8) [22] and followed the cell growth. As shown in Figure 3A & 3B, cell growth was severely inhibited with 100 nM ibr in cells transfected with WT *BTK (*dark blue vs. light blue*)*, whereas continued cell growth was observed in *BTK*^T316A^- or *BTK*^C481S^-transfected cells (Figure 3A & 3B, red vs. orange), demonstrating that the novel T316A mutation generates a level of ibr resistance that is comparable to the C481S mutation. These results were confirmed with the BrdU incorporation assay that showed ibr inhibition of cellular proliferation was significantly lost in cells bearing *BTK*^T316A^ compared to cells bearing WT-BTK (Figure 3C, 31.8% vs 9.71%).

### T316A mutation functionally confers ibr resistance at the molecular signaling level

**Figure 4:**
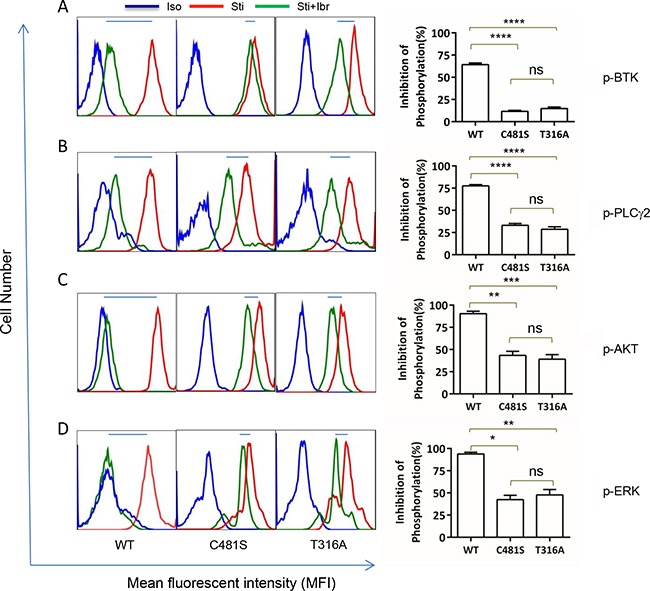
T316A mutation functionally confers ibr resistance at the molecular level Intracellular protein phosphorylation was measured at day 4 after TMD8 transfection. Iso, isotype control. Sti, Cells were stimulated with anti-IgG/IgM antibodies for 10 min before analysis. Sti+Ibr, Cells were treated with 100 nM ibr for 1 hour before stimulation with anti-IgG/IgM antibodies for 10 minutes. Left panels, representative analyses. Right panel, aggregate data for three repeat analyses. Inhibition of phosphorylaton (%) is calculated as (F_Sti_-F_Sti+Ibr_)/ (F_Sti_-F_iso_)x100%, where F denotes mean fluorescent intensity of 10,000 events. Data were analyzed using one-way ANOVA test and graphed with prism 5 GraphPad. **A.** p-BTK (Y223), **B.** p-PLCγ2 (Y759), **C.** p-AKT (S473), and **D.** p-ERK (T202/Y204).

To further understand the molecular mechanisms underlying ibr-resistance caused by *BTK*^T316A^, We measured activity of several key players in the BCR and downstream signaling pathways with phospho-flow assays. Shown in Figure 4A,while p-BTK (Y223) in WT-transfected cells was markedly inhibited by ibrutinib, there remained a significant level of p-BTK in either the C481S or T316A-bearing cells (Figure 4A. compare shifts from red to green, WT vs. C481S vs. T316A column). The degree of phosphorylation inhibition following ibr treatment was significantly less in C481S and T316A mutant cells than in WT cells (Figure 4A right panel, bar graph). When PLCγ2, the substrate of BTK kinase was measured, we observed a similar pattern. The degree of p-PLCγ2 inhibition by ibr was significantly diminished in both C481S and T316A-mutant cells compared with WT cells (Figure 4B). Moreover, resistance to ibr inhibition was reflected further downstream in p-AKT and p-ERK (Figure 4C&4D). Collectively, results from these multiple assays corroborate the conclusion that BCR and downstream signaling pathways were not effectively inhibited by ibr in T316A mutant cells. Together with the cellular experiments of transfected cells (Figure 3), our data firmly established that the new *BTK*^T316A^ mutant is as capable as *BTK*^C481S^ to confer ibr resistance from a functional perspective at both cellular and molecular levels.

## DISCUSSION

In this study, we identified a novel BTK SH2 mutation in a CLL patient. This is the first time BTK mutations outside the kinase domain have ever been reported. With deep sequencing, the mutation was detected only in ibr-relapsed samples, but not in the pre-ibr LN specimen with visibly apparent large cells. The mutation was detected at a high frequency (75%) in the last pleural effusion sample that contained approximately 40% of large Richter transformed cells admixed with small CLL cells suggesting both large and small cells carry the *BTK^T316A^* mutation. Thus, it remains unclear whether the BTK mutation contributes to the process of Richter transformation. A previous report suggests that *BTK* mutations are not associated with RT as only 2 of 9 such patients carried *BTK* or *PLCG2* mutations after ibr treatment [3].

Summarizing all 14 CLL patients reported so far, *BTK* mutations are only detected in patients exposed to ibr. There is not a single case that *BTK* mutations were identified in patients who have not received ibr [2, 3, 6]. However, it remains possible that the mutations are present in minuscule clones prior to therapy. In-silico computational models predict that mutant cells may be present at a frequency of 1/10^6^-1/10^8^ and undergo subsequent clonal selection and expansion with the pressure of the drug [23]. Other mechanisms for ibr resistance may also exist as some ibr-relapsed CLL patients do not carry *BTK*/*PLCG2* mutations [24].

Regarding mutation detection in ibr-relapsed patients, implications of our discovery are two-fold: 1) For management of ibr relapsed/resistant CLL, mutation detection of BTK should include all exons, not just those encoding the kinase domain; and 2) The possibility of a minute mutant clone evolving during ibr treatment entails a highly sensitive technique for clinical early detection. These requirements make conventional techniques such as Sanger sequencing or allele-specific PCR not appropriate. Deep targeted sequencing of *BTK* and other genes involved in ibr-resistance is perhaps the most suitable technology at the present time. Early detection of low-abundance resistance mutation is essential as alternative treatments such as allogeneic transplantation or clinical trials may be considered before overt clinical relapse when the disease is difficult to manage [3, 4].

Unlike other *BTK* kinase domain mutations (C481S, T474A, and L528W) that may confer drug resistance through either attenuating or directly inhibiting ibr binding, T316A is located in the SH2 domain, distant from the drug binding site. The SH2 domain is responsible for interacting with phosphotyrosine-containing peptide substrates (Figure 2D). The major binding partner of BTK SH2 domain is B-cell linker protein (BLNK). Since interaction between BTK and BLNK is essential for the phosphorylation and activation of the downstream kinase substrate PLCG2 [21], T316A substitution would be predicted to prevent key contact to phosphotyrosine, thus may lessen affinity of BTK for BLNK or other BTK partner proteins. However, the activity of PLCG2 upon stimulation seemed to be comparable at a similar level across cells bearing either WT or mutant BTK proteins (Figure 4B, compare red vs blue peaks), thus we had no evidence suggesting that the mutations, either C481S or T316A, changed the kinase activity of BTK. Regarding ibr binding, it does remain possible that a distal mutation in SH2 could change the three dimensional structure of the protein in such a way that ibr binding is disrupted.

Additional studies will be required to further understand how the mutation impacts on ibr binding from the structural and biochemical perspective.

Nonetheless, our functional studies at both cellular and molecular levels demonstrate that T316A confers ibr resistance to a similar extent as C481S, providing insight into the diversity of ibr resistance. Notably, the mutation mechanisms between *BTK*-ibr in CLL parallel that of BCR/ABL1-imatinib in chronic myeloid leukemia. ABL1 mutations which confer imatinib resistance also involve both kinase and SH2 domain. Thus, the novel mutation mechanism described herein may be exploited for the rational design of next-generation BTK inhibitors resembling the evolution of ABL inhibitor development.

## MATERIALS AND METHODS

### Patient samples

Research was conducted on diagnostic residual samples in accordance with the Declaration of Helsinki and institutional IRB policies. Serial samples at four time points throughout patient's disease course were studied.

### Purification of CD19+ B cells and DNA isolation

For blood and bone marrow, mononuclear cells were isolated following red cell lysis. B-cells were then enriched and purified using Dynabeads^®^ CD19 pan B kit (Life Technologies, Oslo, Norway) with 90% purity assessed by flow cytometry. DNA was isolated using QIAamp DNA mini kit for S1 and S3 samples and QIAamp DNA FFPE Tissue kit for S2 and S4 samples.

### Next generation sequencing (NGS)

A hybrid capture panel with 1,200 cancer-associated genes (Onco1K), developed per CLIA standards was used to detect genomic alterations in S1 (Dx) and S3 (Ibr failure) with an average sequencing depth of 420x (S2 and S4 did not have sufficient DNA for this assay). Libraries were prepared from 200 ng of isolated DNA (Kapa Biosystems), subject to hybrid capture (Roche Nimblegen) and sequenced via HiSeq 2500 (Illumina) with 21.9 and 19.3 million read pairs respectively. The *BTK* mutated position was sequenced at a depth of 800x in S1 and 400x in S3. Targeted sequencing was performed for S1-S4 at an average depth of 2,300x using a 17-gene amplicon-based CLL panel. Deeper targeted-sequencing of *BTK* was performed for S1 and S2 samples at depth of 6200x and 3700x respectively using the CLL panel. In this assay, multiplex PCR was done with 10 ng DNA, libraries were prepared (Kapa Biosystems), and sequenced via MiSeq (Illumina).

### NGS data analysis

Data analysis for both panels was performed on a HIPAA-compliant high performance computing system (Center for Research Informatics, The University of Chicago) using in-house developed bioinformatics pipelines, with variant detection performed at a threshold of 10% mutant allelic fraction (MAF) for Onco1K and 5% for CLL Panel. ∼22 million read pairs were sequenced per library. The informatics pipeline included quality checks (FastQC [http://www.bioinformatics.babraham.ac.uk/projects/fastqc/]) and adapter trimming, followed by alignment using Burrows-Wheeler Aligner, [8, 9] and Indel-realignment. The point mutations and small indels were detected using a combination of Samtools pileup [9] and an in-house pileup analyzer toolkit. Amplicon Indel Hunter was used for indel detection in CLL Panel [10]. The resulting mutations were annotated using Alamut (http://www.interactive-biosoftware.com/). Additional filters were used on the annotated files based on 1000G frequencies (to remove inherited SNPs), SIFT predictions and coding effects to return a final list of somatic mutations (54 in S1 and 56 in S3) by large panel.

### Sanger sequencing

The presence of *BTK*^T316A^ mutation was confirmed using Sanger sequencing. Primers (Forward primer: 5′GAGACAGAGGAAGTGGGACG 3′ Reverse primer: 5′GCACCACTTCCTCCTACAGA 3′) were designed to amplify exon 11 of *BTK* encompassing the mutation. The polymerase chain reaction (PCR) product of 217 bp was subjected to Sanger sequencing.

### Structural analysis

PyMOL was used to analyze the domain structures of BTK, including PH domain and TH domain (PDB code=1BTK), SH3 domain (1QLY), SH2 domain (2GE9), and kinase domain (3GEN).

### Generation of *BTK* C481S and T316A mutant constructs

*BTK* WT cDNA clone in pCMV6 expression vector was purchased from ORIGENE (Rockwille, MD USA). *BTK*^C481S^ and *BTK*^T316A^ mutant vectors were generated using QuikChange II Site-Directed Mutagenesis Kit (Agilent Technologies, Ceder Creek, TX, USA) following manufacturer's instructions. The identity of the mutant constructs was confirmed by Sanger sequencing.

### Cell culture and transfection

TMD8 cells were maintained in RPMI1640 at 37°C with 10% fetal calf serum (Mediatech Inc, Manassas, VA, USA), 100 U/mL penicillin and 100 μg/mL streptomycin (Fisher Scientific, Fairlawn, NJ, USA). For cell transfection with *BTK* WT, *BTK*^C481S^ and *BTK*^T316A^ mutant constructs, Amaxa Nucleofection technology was applied according to the manufacturer's protocols (Amaxa, Cologne, Germany; kit V, Program U-13). To enhance the cell survival following transfection, TMD8 cells were co-cultured with bone marrow stromal cell line NKTert cells in a 24-well plate for the first 24hr. Cells were subsequently transferred into a new plate and ibr or vehicle was then added into the culture. Cell viability was determined with Muse™ Count & Viability kit using Muse Cell Analyzer (Millipore, Hayward, CA).

### Cell cycle analysis

*BTK* WT or mutant-transfected TMD8 cells were treated with ibr for 72 hrs at indicated doses. Cells were then exposed to 10 μM BrdU for 2 hrs followed by cell cycle analysis according to the manufacturer's instructions using BrdU Flow Kit (BD Pharmingen, San Diego, CA, USA). Flow cytometric analysis was performed using 4-laser BD LSR II using FACSDiva and FlowJo software.

### Intracellular phospho-flow staining and flow cytometry analysis

Intracellular phospho-flow assay was conducted as described previously [11–15]. Briefly, 1×10^6^ *BTK* WT- or mutant-transfected TMD8 cells were treated with 100 nM ibr for 1 hr at 37°C and followed by stimulation of 5 μg/mL of goat F(ab')2 anti-human IgM/IgG (Southern Biotech, Birmingham, AL USA) for 15 min. Cells were then fixed in 4% formaldehyde for 10 min and permeabilized with 100% methanol for 20 min, and were subjected to staining with Alexa Fluor^®^ 647-anti-phospho-AKT (Ser473), Alexa Fluor^®^ 488 anti-phospho-ERK1/2 (Thr202/Tyr204) (Cell Signaling, Billerica, MA USA), PE-anti-phospho-PLCg2 (Tyr759), and PE-anti-phospho-BTK (Tyr223) (BD Bioscience, Franklin Lakes, NJ). Flow cytometry was performed with BD™ LSR II flow cytometer and data analyzed with FlowJo v10.

## SUPPLEMENTARY MATERIALS FIGURE AND TABLES




