# Supplementary material for: Identification of a structurally novel BTK mutation that drives ibrutinib resistance in CLL
Source: Oncotarget. 2016 Sep 10;7(42):68833–41. doi: 10.18632/oncotarget.11932 (PMC5356593; doi:10.18632/oncotarget.11932)
Supplement: Supplementary file 1 [file oncotarget-07-68833-s001.pdf]

Identification of a structurally novel BTK mutation that drives ibrutinib resistance in CLL

SUPPLEMENTARY FIGURE AND TABLES

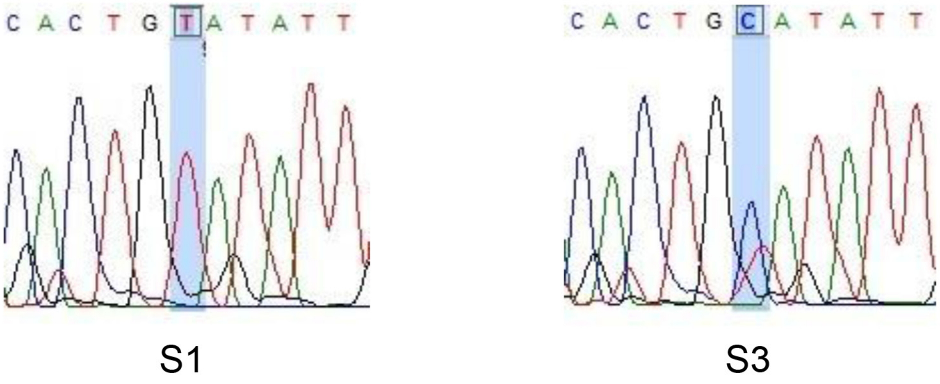

Supplementary Figure S1: Sanger sequencing results. Sanger sequencing confirmation of *BTK* T316A mutation in S1 (CLL diagnosis) and S3 (Post-ibr CLL relapse). Primers were designed to amplify exon 11 of BTK. The *BTK* mutated position is highlighted in blue.

Supplementary Table S1: List of 17 genes in the CLL panel

ATM	CXCR4	MYD88	SF3B1
BCOR	FAT3	NOTCH1	SPEN
BIRC3	FBXW7	NRAS	TP53
BRAF	KRAS	PLCG2	XPO1
BTK			

All the exons in these 17 genes were sequenced in S1-S4 at an average depth of 2300x using this panel. Deeper targeted re-sequencing of BTK was performed for S1 and S2 at depth of 6200X and 3700X respectively.

Supplementary Table S2: Comparison of variant frequencies across S1, S2, S3 and S4, as determined by the CLL Panel. All potential somatic variants found in at least one of 4 samples and their annotations are shown. The last column shows a bar graph of the allele frequencies in each of the 4 samples.

See Supplementary File 1
